# Chronic Inflammation in Skin Malignancies

**DOI:** 10.5334/1750-2187-11-2

**Published:** 2016-05-05

**Authors:** Lihua Tang, Kepeng Wang

**Affiliations:** Department of Dermatology, 303 Hospital of People’s Liberation Army, Nanning, Guangxi Province, China; Department of Immunology, School of Medicine, University of Connecticut Health Center, Farmington, Connecticut, USA

**Keywords:** inflammation, skin cancer, cytokine, cancer immunotherapy

## Abstract

Chronic inflammation is linked to the development and progression of multiple cancers, including those of the lung, stomach, liver, colon, breast and skin. Inflammation not only drives the oncogenic transformation of epithelial cells under the stress of chronic infection and autoimmune diseases, but also promotes the growth, progression and metastatic spread of cancers. Tumor-infiltrating inflammatory cells are comprised of a diverse population of myeloid and immune cell types, including monocytes, macrophages, dendritic cells, T and B cells, and others. Different myeloid and lymphoid cells within tumor microenvironment exert diverse, often contradicting, effects during skin cancer development and progression. The nature of tumor-immune interaction determines the rate of cancer progression and the outcome of cancer treatment. Inflammatory environment within skin tumor also inhibits naturally occurring anti-tumor immunity and limits the efficacy of cancer immunotherapy. In this article we aim to give an overview on the mechanism by which inflammation interferes with the development and therapeutic intervention of cancers, especially those of the skin.

## Introduction

Inflammation is characterized by the infiltration of plasma and leukocytes to tissues that undergo disrupted homeostasis [1]. The causes of inflammation range from pathogenic infection, tissue injury to tissue stress and malfunction [1]. Inflammatory process is critical for normal physiological responses against infection and tissue damage, and promotes the clearance of invading pathogens and the regeneration of damaged host tissues. Inflammation is also important for maintaining homeostasis and monitoring stress signals that arise with tissue malfunction [1,2]. However, the process of inflammation may bring detrimental side effects to the host, depending on the nature, duration and magnitude of inflammatory response elicited during infections and diseases. Examples of such side effects include allergies, autoimmune diseases, and life-threatening immune responses induced by viral and bacterial infection in humans [3,4,5,6]. Inflammation is also recognized as one important player in the entire course of carcinogenesis [7,8]. Different myeloid and lymphoid cells infiltrate into tumor stroma and exert divergent, even contradicting effects on the growth, progression and metastatic spread of cancers [7,8]. In this review we will summarize our current understanding on the nature of immune-cancer interaction, focusing primarily on skin malignancies.

There are four major types of skin malignancies: basal cell carcinoma, squamous cell carcinoma, melanoma and nonepithelial skin cancers [9]. Among them, melanoma is the most deadly form of skin cancer and contributes to 10,000 deaths per year in the United States [10]. About 132,000 new cases of melanoma arise globally each year, leading to vast majority of skin cancer-related deaths [11]. Risk factors of skin carcinogenesis include chronic cutaneous inflammation, viral infection, ultraviolet radiation (UVR), and other inflammation-inducing agents and traumas [12,13]. UVR promotes the transformation of skin cells by damaging cellular DNA. The major DNA damage products generated through UVR exposure are cyclobutane pyrimidine dimers and pyrimidine [4,5,6] pyrimidone [14]. Damaged DNA is typically repaired by the nucleotide excision repair pathway, whereas defective repair of the damaged DNA results in cancer predisposition [15]. UVR also serves as a link between skin cancer and inflammation, as its exposure alters immunological functions in the skin [16]. For example, exposure to UV light results in the upregulation of COX-2 protein in keratinocytes and increased production of prostaglandin E2 (PGE2), which leads to cutaneous tissue inflammation [17]. UV exposure also adversely affects skin immune system by suppressing the function of antigen-presenting cells, inducing the expression of immune-suppressive cytokines and modulating contact and delayed-type hypersensitivity reactions [18]. The suppression on adaptive immunity by UVR has been proposed to contribute to the evasion of skin cancer cells from immune surveillance [18]. UVR therefore promotes skin carcinogenesis through both direct action on skin cells and indirect modulating effect on local microenvironment that is shaped by the process of chronic inflammation and immune response.

Chronic inflammation has been recognized a driving force for epidermal cell transformation and malignant progression. In this article, we aim to summarize our current knowledge on the role of inflammatory signaling in different types of cancers, followed by a more detailed description on the role of inflammation in skin cancer development.

## Chronic inflammation promotes cancer development and progression

The link between inflammation and cancer has long been suspected due to the pioneering work of Rudolf Virchow over 150 years ago [19]. Since that point these tumor-infiltrating cells have been suspected to play a role in cancer development and progression. Experimental evidence linking inflammatory and immune cells to cancer development, however, was only provided in the past decade by the use of mouse models of cancers [7,8,20]. Tumor infiltrating myeloid and lymphoid cells can either promote or inhibit cancer development, depending on the nature of the immune-cancer interaction [7,20,21]. Through the production of cytokines, chemokines and extracellular enzymes, tumor infiltrating immune cells may serve as tumor promoter by supporting tumor cell proliferation and inhibiting programmed cell death [7,8,20]. On the other hand, innate and adaptive immune cells recognize tumor-specific antigens and molecular patterns and actively destroy transformed cells [22]. In addition to the direct tumor-immune interaction, different branches of immune cells also crosstalk within the tumor microenvironment and regulate their counterparts’ recruitment and activity. Immune cells also signal to other stromal cells in the tumor, such as fibroblasts and endothelial cells, to promote the production of cytokines and chemokines and regulate oxygen and nutrient supply to tumor cells [23,24]. The eventual outcome of this complicated network of regulation is the formation of a unique tumor microenvironment that has profound impacts on the development, progression, and metastatic spread of cancers. Shaping the tumor microenvironment by immune cells also plays an important role in determining the outcome of anti-cancer therapy in humans.

**Figure 1:**
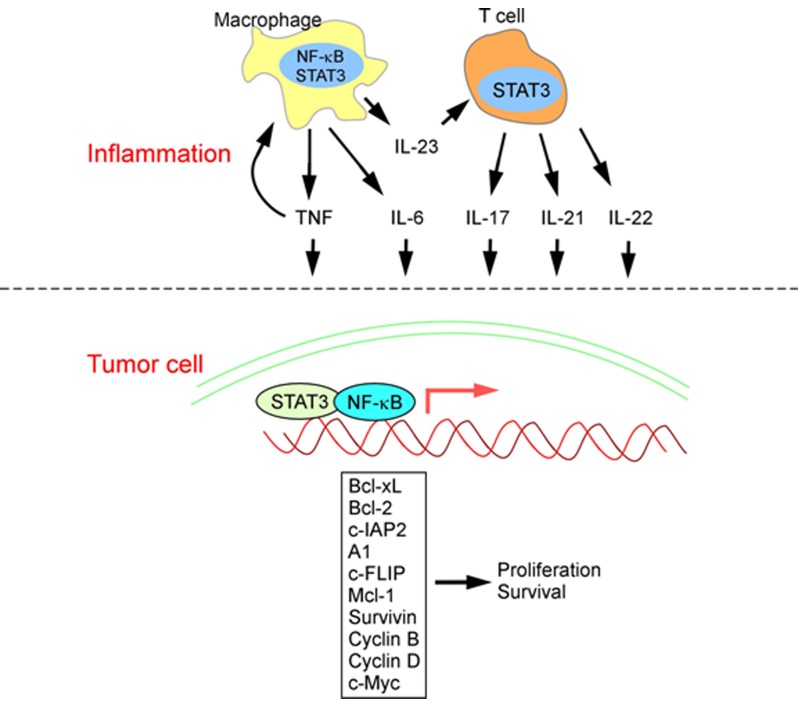
Inflammation promotes tumor growth and survival. Tumor-infiltrating myeloid cells and lymphocytes produce inflammatory cytokines including TNFα, IL-6, IL-17, IL-21, IL-22 and IL-23. TNFα activates NF-κB in myeloid cells and stimulates tumor-associated inflammation. IL-23 signals to T lymphocytes and other immune cells to stimulate the production of IL-17, IL-21 and IL-22. TNFα, IL-6, IL-17, IL-21 and IL-22 activate STAT3 and NF-κB signaling in transformed epithelial cells. Activated STAT3 and NF-κB transcribe genes that support cell proliferation and survivial, thereby increases the rate of cancer growth, progression and metastatic spread.

Chronic inflammation contributes to about 20% of all human cancers [7]. Examples of such association include hepatitis B and C virus infection with liver cancer [25]; *Helicobacter pylori* colonization with gastric cancer [26]; ulcerative colitis [27,28] and Crohn’s disease [29,30] contributing to colorectal cancer; and smoking [31,32] and asbestos exposure [33,34] with lung cancer. Under normal conditions, inflammation serves as a mechanism of host defense and tissue regeneration following pathogen infection or tissue damage. However, under persistent infection or injury, chronic inflammation drives the transformation of cancer-originating cells by producing reactive oxygen species (ROS) and reactive nitrogen intermediates (RNI) that are capable of inducing DNA damage and genomic instability [35,36]. In addition, tumor-infiltrating myeloid and lymphoid cells produce cytokines that signal to transformed cells and support their growth and survival. These pro-tumorigenic cytokines include interleukin (IL)-6, IL-11, IL-21 and IL-22 that activate the STAT3 transcription factor; TNFα, IL-1 and IL-18 that activate NF-κB; and the IL-23 to IL-17 axis of inflammation that activates both STAT3 and NF-κB in tumor cells [37,38] (Figure 1).

NF-κB and STAT3 are essential for inflammation-promoted cancer development [39,40,41,42]. NF-κB signaling plays important roles in normal physiology and immunity. Activation of NF-κB depends on the phosphorylation of the IκB protein by the IKK complex comprised of IKK-α, IKK-β and IKK-γ. Phosphorylation of IκB leads to its poly-ubiquitination and proteasomal degradation, thereby releasing NF-κB to cellular nucleus for transcriptional activation of its target genes [43,44]. NF-κB signaling promotes cancer development by its activity within both cancer cells and immune cells [45]. Activation of NF-κB in immune cells results in the expression and production of multiple pro-inflammatory cytokines, including TNFα, IL-1, IL-6, IL-17 and IL-23, which promote cancer development in multiple mouse models [37,45,46,47,48,49]. Activation of NF-κB in cancer cells enhances their survival as a result of the upregulation of anti-apoptotic genes such as Bcl-xL, Bcl-2, c-IAP2, A1 and c-FLIP [50,51].

STAT3 can be activated in cancer cells by multiple cytokines and growth factors, best known for IL-6 and its family members [40]. Activation of STAT3 requires engagement of extracellular ligands (e.g. IL-6) to their cognate receptors, followed by receptor dimerization and activation of JAK kinases. JAKs subsequently phosphorylate the tyrosine 705 residue on STAT3 that permits its dimerization, nuclear translocation and target gene activation [52]. STAT3 activation in cancer cells results in enhanced cell proliferation and survival. The increase in cancer cell proliferation is mediated by STAT3-activated production of Bcl-xL, Bcl-2 and c-IAP2, which are also activated by NF-κB [53,54,55,56]. Mcl-1 and Survivin are also upregulated by STAT3 signaling and promote cancer cell survival [53,54,55,56]. STAT3 also promotes cell cycle progression by transcribing genes encoding c-Myc and cyclins B and D [54,55,56]. Taken together, inflammatory environment within tumors promotes cancer development by activating NF-κB and STAT3 signaling and upregulating pro-survival and cell cycle-driving genes (Figure 1).

## Chronic inflammation that accelerates skin carcinogenesis

The skin is a unique epithelial tissue that covers our body and provides physical and biological surface protection [57]. It contains three layers: epidermis, dermis and subcutaneous layer [58,59]. The epidermis is the most outer layer composed of keratinocytes and melanocytes. Keratinocytes originate from the basal layer of the epidermis and migrate toward the surface, where they are gradually shed and replaced by newer cells [58]. Melanocytes are scattered throughout the basal layer of the epidermis and produce melanin that determines our skin color [60,61]. The main function of melanin is to block the penetration of UVR from the sunlight, which damages DNA and induces skin tumorigenesis [60,61]. The epidermis also contains residential macrophages called Langerhans cells that defend the body against foreign microbial infection [62]. Below the epidermis is the dermis that contains fibrous and elastic tissue that gives the skin its flexibility and strength. The dermis also contains nerve endings, sweat glands, blood vessels and hair follicles [57,58,59]. Further below is the subcutaneous layer that insulates our body from heat loss and stores energy in the form of fat [57].

With the use of mouse models of skin cancer, it is now clear that pro-inflammatory immune cells play important roles in skin cancer development (Figure 1) [12,13,63]. One of the first studies demonstrating the importance of inflammation in skin cancer pointed to a tumor-promoting role of TNFα [48,49]. TNFα is known to promote autoimmune inflammation in the skin, including psoriasis [64,65]. Mice harboring genetic ablation of *Tnfa* were resistant to skin tumor development that was initiated by DMBA and promoted by TPA protocol [49]. The same resistance was observed when mice were applied repeated doses of DMBA without TPA [49]. TNFα also promotes UVR-induced cutaneous squamous cell carcinomas in PKCε transgenic mice [48]. TNFα signals to both tumor cells and their surrounding stromal cells during skin cancer development [49]. TNFα signaling in early stage skin cancer activates transcription factor AP-1 and promotes the production of GM-CSF, MMP-3, 7 and 9 [48,66]. Activation of NF-κB transcription factors by TNFα promotes the upregulation of c-FLIP and enhances skin tumor cell survival, and confers their resistance to RAF inhibitor treatment [48,67]. Ablation of either TNFR1 or TNFR2 resulted in reduced skin cancer development, with TNFR1 contributing to a larger share of the tumor-promoting effect [68]. Consistent with the pro-tumorigenic role of TNFα in the skin, administration of a TNFα-neutralizing antibody to mice significantly reduced skin tumor development [69].

In addition to TNFα, IL-1/MyD88 signaling has also been attributed to keratinocyte transformation and carcinogenesis [70]. Ablation of the receptor for IL-1 cytokine (IL-1R), or its downstream signaling adaptor molecule MyD88, resulted in reduced topical carcinogenesis that was induced by the DMBA/TPA protocol [70]. Adoptive transfer of WT or MyD88-deficient bone marrow cells showed that MyD88 is needed in both hematopoietic and radio-resistant cells during skin carcinogenesis. Targeted ablation of MyD88 in basal keratinocytes reduced skin tumor load by half, further confirming a direct tumor-promoting role of IL-1R/MyD88 signaling within skin cells [70]. On the other hand, ablation of the MyD88 adaptor protein in all hematopoietic cells also resulted in significant reduction in skin tumor load [70]. Activation of keratinocytes by IL-1R/MyD88 signaling results in the activation of NF-κB and increased production of cytokines and chemokines that have been shown to promote skin carcinogenesis, including TNFα, CXCL1, CSF2 and MMP9 [70]. CXCL1 binds to its cognate receptor CXCR2 on keratinocytes and contributes to tumor formation and metastatic spread [71]. The role of CSF2 (GM-CSF) in skin cancer is context-dependent. Over-expression of CSF2 in the skin resulted in increased tumor burden in a mouse model of squamous cell carcinoma, whereas expression of its antagonist inhibits the rejection of B16 melanoma cells [72], suggesting a dual role of CSF2 in regulating pro- and anti-tumor immunity. Regarding the source of IL-1 cytokine in skin cancer, it has been shown that UV challenge or TPA stimulation leads to production of IL-1α by keratinocytes [73,74]. Activation of K-Ras, a potent oncogene that drives the development of multiple cancers [75], in transformed skin cells resulted in production of IL-1α, which signals in an autocrine manner through IL-1R/MyD88/NF-κB pathway to synergize with K-Ras for the oncogenic progression of skin cancer [70].

The role of IL-6 family cytokines has been extensively studied in multiple mouse models of cancers. Although the *in vivo* test on IL-6 in mouse models of skin cancer is lacking at this point, cell line-based studies have shown that IL-6 plausibly promotes skin tumor growth through activation of the STAT3 transcription factor [76]. IL-6 can be produced by keratinocytes that are stimulated by UVR or TPA exposure, thereby akin to IL-1, signals in an autocrine manner in transformed keratinocytes [73,74]. There are only limited studies on the involvement of the other IL-6 family cytokines in skin cancer, though we now know that IL-11 is over-expressed in skin tumors and promotes tumor development through the activation of STAT3 [77]. Consistent with its role in mediating the signaling of IL-6 and its family members, STAT3 has been shown to drive both the initiation and promotion phases of epithelial carcinogenesis [77,78]. Epidermal specific ablation of STAT3 resulted in dramatically reduced skin tumor load, in both oncogene- and UVR-driven mouse models of skin cancers [77,79]. One of the targets of STAT3 signaling in skin cancer development is Bcl-xL, whose ablation resulted in marked reduction in skin tumor load [80]. Similarly, forced expression of another STAT3 target, Survivin, in the skin led to increased chemical-induced carcinogenesis and decreased tumor regression [81]. In addition to supporting primary tumor growth, STAT3 also drives metastatic spread of melanoma by inhibiting cell apoptosis during anoikis (anchorage-independent cell death) [82]. Activation of STAT3 by IL-6 in melanoma cells promotes the expression of Twist and N-cadherin proteins, which are markers of epithelial-to-mesenchymal transition (EMT) [83].

The IL-23/IL-17 axis of inflammation contributes significantly to the development of multiple cancers including that of the skin. IL-23 belongs to the IL-12 family of heterodimeric cytokines. IL-23 shares the 40 kD subunit with IL-12, and has its unique 19 kD subunit encoded by the *Il23a* gene [47,84,85]. IL-12 is comprised of a 35 kD (encoded by the *Il12a* gene) and a 40 kD (encoded by the *Il12b* gene) subunit [47,84,85]. Ablation of *Il23a* resulted in marked reduction in DMBA/TPA-induced skin tumors, suggesting a strong tumor-promoting role of IL-23 in the skin [86]. It is intriguing that deletion of IL-12 resulted in marked increase in skin tumorigenesis, opposite to that of IL-23 [86]. Mice harboring deletion of the common p40 subunit that is shared by IL-23 and IL-12 also failed to develop skin tumors [86].

IL-23 is mainly produced by activated macrophages in response to engagement of Toll like receptors (TLRs) and subsequent activation of NF-κB and STAT3 transcription factors [87,88,89]. IL-23 is important for the expression of another cytokine IL-17 by phenotypically stabilizing and inducing the expansion of IL-17 producing T cells (Th17 cells) or through activation of innate lymphoid cells (iLC) and γδ T cells together with IL-1 [90,91,92,93]. IL-17, in conjunction with IL-22 that are both produced by Th17 cells, supports the development of skin cancer by activating STAT3 in tumor and stromal cells and promoting the infiltration of myeloid cells into the tumor microenvironment [94,95,96]. In addition to IL-22, IL-6 and IL-11 also drives the malignant progression of skin cancer cells through the activation of STAT3 and upregulation of inflammatory and angiogenic factors [76,77].

## Cytotoxic T cell-related cytokines suppress skin cancer development

Though in many cases cancer-associated inflammation promotes the development of skin malignancies, our immune system does provide protection against cancer development through both innate and adaptive immunity [97,98]. These naturally occurred anti-cancer immunity not only limits the rate of carcinogenesis in humans but also provides the ground for cancer immunotherapy. Among anti-tumor immune cells and cytokines are IL-12 and interferon-gamma (IFN-γ) that play central roles in limiting skin cancer development. IL-12 is a heterodimeric cytokine that is composed of a p35 subunit and a p40 subunit [47,84,85]. The p40 subunit is shared with IL-23, which has been shown to promote skin tumorigenesis (86). Unlike that of IL-23, ablation of IL-12 by knocking out the p35 subunit resulted in marked increase in mouse skin tumor load, suggesting an anti-tumor role of IL-12 [86]. Importantly, when the common p40 subunit was knocked out in mice under DMBA/TPA protocol for skin cancer induction, these mice developed few skin tumors, similar to that of IL-23 knockout [86]. These results suggest that the effect of tumor promotion by IL-23 dominates early phase skin carcinogenesis [86].

Consistent with the known role of IL-12 in activating cell-mediated immunity through CD4^+^ type 1 T helper cells (Th1 cells) and CD8^+^ cytotoxic lymphocytes (CTLs), ablation of CD8^+^ cells in mice resulted in marked increase in skin tumor load following DMBA/TPA protocol [99]. Similarly, mouse models of skin cancer also showed a critical role for IFN-γ in anti-tumor immunity [100,101]. Mice lacking the receptor for IFN-γ, or its downstream signaling mediator STAT1, are prone to chemical carcinogen mediated sarcoma induction [102,103]. IFN-γ is produced by Th1 cells, CTLs and γδ T cells and potently activates cell-mediated immunity against tumor cells [100,101,104]. In response to IFN-γ signaling, natural killer cells (NK cells) and CTLs recognize tumor-specific surface traits and eliminate transformed cells [100,101]. Of special interest are the discovery of immune modulatory mechanisms that limit T cell anti-tumor activity, and the invention of novel therapies targeting T cell modulating or co-stimulatory pathways for the treatment of melanomas and other solid tumors [105,106]. These progresses have been reviewed extensively in recent publications and we will not go into details here.

## Immune-modulating cytokines in skin cancer

In addition to pro- and anti-tumorigenic cytokines that we introduced in the previous sessions, immune modulatory cytokines also play important, sometimes controversial roles in skin cancer development and therapy. These molecules include IL-10 and transforming growth factor-β (TGF-β) that are both produced by regulatory T cells (Treg) and other immune and stromal cells in the tumor.

IL-10 is produced by Treg cells, macrophages, dendritic cells (DC) and epithelial cells, and dampens inflammatory and immune responses [107,108]. UV irradiation induces Treg cell expansion in the skin, whereas Treg suppresses Th1-driven immunity against skin cancer through the production of IL-10 [18,109]. IL-10 knockout mice are resistant to UV-induced skin carcinogenesis [109]. Adoptive transfer of UV-induced regulatory T cells from IL-10-deficient mice failed to suppress Th1 response against skin cancer [109]. These results suggest that IL-10 mainly limits anti-tumor adaptive immunity during skin cancer development.

TGF-β is another immune modulating cytokine that is produced by Treg cells in tumor microenvironment [110,111]. Intriguingly, TGF-β also promotes the differentiation of naïve T cells into Treg cells in the periphery, thereby forming an auto-enforcing loop for the suppression of autoimmunity and prolonged inflammation in animals and humans [112]. The role of TGF-β in cancer is manifested by its function in limiting both tumor-promoting inflammation and anti-tumor immunity, thereby the outcome of its ablation depends on the quality of immune response within tumor microenvironment [113]. TGF-β inhibits the proliferation of keratinocytes and its inactivation (by targeted expression of a dominant negative form of TGF-β receptor TGFBR-2) resulted in increased keratinocyte number and thickened skin in mice [114]. Development of papilloma was also accelerated in mice lacking TGF-β signaling and persisted after the cessation of TPA treatment and progressed to squamous cell carcinoma with increased angiogenesis and metastasis [115]. Similarly, skin-specific ablation of TGFBR-2 resulted in enhanced cutaneous carcinogenesis that was induced by K-Ras activation or DMBA treatment [116]. Therefore TGF-β suppresses primary skin tumor development by limiting cancer-promoting inflammatory pathways and by its direct action on transformed epithelial cells.

In contrary to its role in limiting primary tumor development, TGF-β promotes the metastatic spread of multiple cancers including that of the skin [117,118]. The major mechanism by which TGF-β promotes metastasis is through its signaling into cancer cells and activation of the epithelial-mesenchymal transition (EMT) process, through which cancer cells acquire enhanced capacity in cell motility and tissue invasion [118,119]. Suppression of adaptive immune response within tumor microenvironment by TGF-β also interferes with the ability of the immune system to eradicate cancer [120]. Depending on the stage of the cancer development and the strategy of intervention, blocking TGF-β signaling can be beneficial, especially in the case of cancer vaccination and immunotherapy.

Taken together, the role of immune modulating cells and cytokines in skin cancers are circumstance-specific. Immune-suppressive activity of IL-10 and TGF-β limits both cancer-promoting inflammation and anti-cancer immunity. Targeting these immune pathways for the prevention and/or treatment of cancers require careful evaluation on their effects on both arms of immunity in cancer, so that pro-tumorigenic inflammation is limited to its minimal level while Th1-lineage adaptive immunity can be maximized for cancer eradication. The list of pro- and anti-cancer cytokines is shown in Table 1.

**Table 1:** Cytokines in skin cancer development.

Cytokine	Model	Effect	Mechanism of Action	References

TNFα	DMBA/TPA; UVR-induced skin cancer	pro-tumor	Activates NF-κB in tumor cells. Enhances tumor cell survival. Exacerbates tumor-associated inflammation.	[48,49]
IL-1	DMBA/TPA; K-Ras activation in skin cells.	pro-tumor	Activates NF-κB in tumor cells. Synergizes with K-Ras to drive cancer progression. Exacerbates tumor-associated inflammation.	[70]
IL-6	Skin cancer cell culture	pro-tumor	Activates STAT3 transcription factor and upregulates Bcl-xL to promote cell survival.	[76]
IL-17	DMBA/TPA	pro-tumor	Activates STAT3 in tumor cells and promotes skin tumor-associated inflammation	[94,95,96]
IL-23	DMBA/TPA	pro-tumor	Activates Th17 cells and upregulates the production of IL-17.	[86]
IL-12	DMBA/TPA	Anti-tumor	Activation of CTLs and NK cells in tumor.	[86]
IFN-γ	Carcinogen-induced sarcoma	Anti-tumor	Activation of CTLs and NK cells in tumor.	[100,101 ]
IL-10	UVR-induced skin cancer	Pro-tumor	Limits Th1 response in tumor.	[109]
TGF-β	DMBA/TPA, melanoma	Pro-tumor/anti-tumor	Inhibits keratinocyte proliferation; limits tumor-associated inflammation; limits cell-mediated immunity against cancer; promotes cancer metastasis.	[115,116,117,118,120]

## A cellular perspective on inflammation and cancer

**Figure 2:**
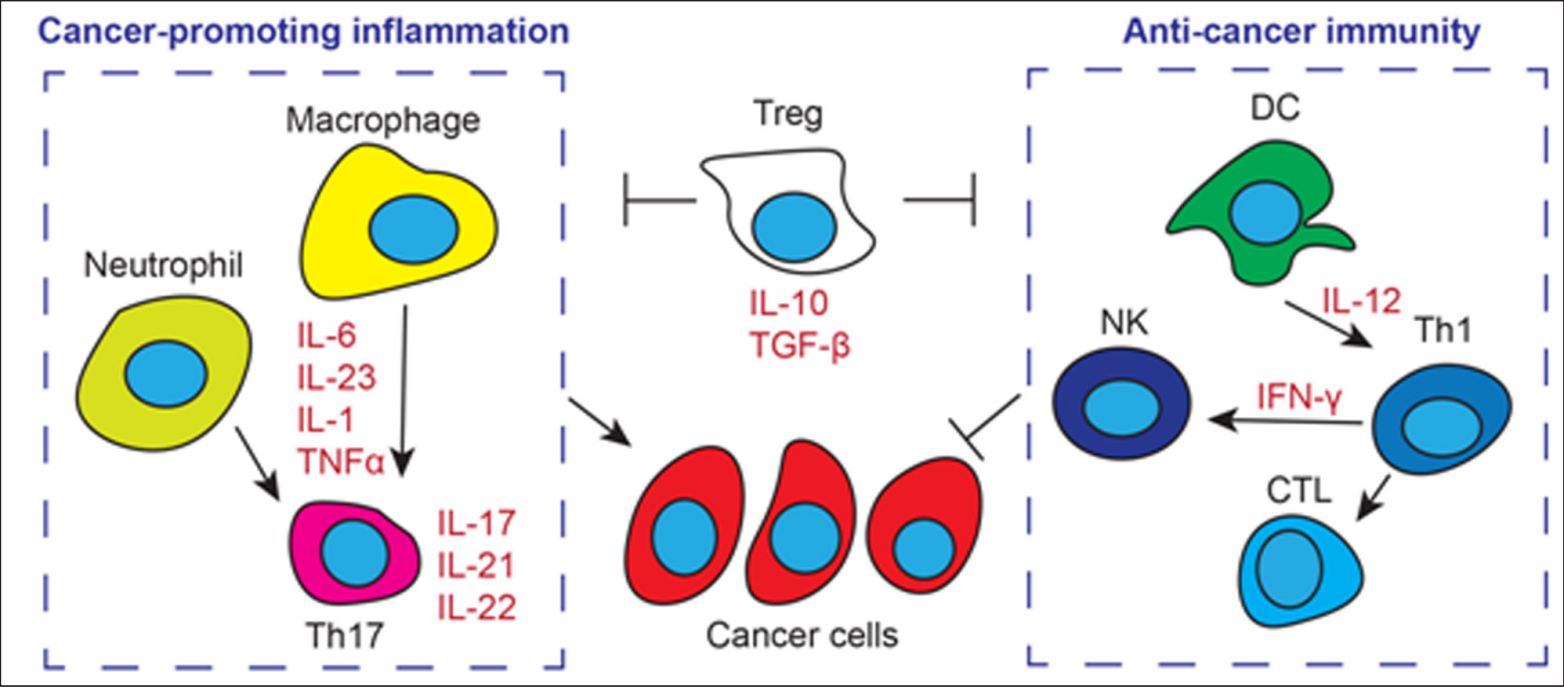
The roles of tumor infiltrating immune cells. Tumor infiltrating macrophages and neutrophils produce IL-6, IL-23, IL-1 and TNFα, and activate Th17 lymphocytes for the production of IL-17, IL-21 and IL-22. These cytokines act on cancer cells to drive their proliferation and survival. Dendritic cells (DC) facilitate T cell-mediated immunity against cancer by IL-12 production and antigen presentation. Th1 cells further activate natural killer (NK) cells and CTLs by secreting IFN-γ. NK cells and CTLs target cancer cells for destruction. Regulatory T cells (Treg) produce IL-10 and TGF-β and inhibit both pro- and anti-cancer immunity by contact dependent and independent mechanisms.

Thus far we have been focusing on cytokines as mediators of immune responses that support or limit cancer development. Cancer-infiltrating immune cells are the major source of these cytokines. There are a variety of myeloid and lymphoid cells that infiltrate tumor stroma, demonstrating the complexity of tumor-immune interacting network. These include natural killer cells (NK cells), CTL, Th1, Th17 and Treg lymphocytes, macrophages, monocytes, dendritic cells (DCs) and other cell types [121]. In a simplified view, tumor infiltrating monocytes, macrophages and Th17 lymphocytes produce cytokines like IL-1, IL-6, IL-17, IL-23 and TNFα, which signal to exacerbate tumor-associated inflammation and activate survival and proliferation machinery in cancer cells [7,8,20,42]. On the other hand, NK cells and Th1/CTL lymphocytes (with the facilitation of antigen-presenting DCs) recognize tumor-specific surface patterns and eradicate cancer cells [22,63,101,105]. Regulatory T cells produce immune modulatory cytokines IL-10 and TGF-β, and exert immune suppression via contact dependent and independent mechanisms [110]. The role of Treg cells in cancer development is stage and context-specific, depending on the relative strength of pro- and anti-tumor immunity in the local environment [105,122] (Figure 2).

## Concluding remarks

The interaction between immune cells and cancer cells has long been speculated, but experimental evidence demonstrating the roles of different myeloid and lymphoid cells in cancer development and prognosis only became available in the last decade or so. We now know that the immune system acts like a double-edged sword that can either promote or inhibit cancer development. The ultimate goal for cancer immunological study is to achieve a treatment outcome where cancer-promoting cytokine signaling (TNFα, IL-1, IL-6, IL-17 and IL-23) is blocked to slow down cancer cell growth and reduce their survival and therapy resistance (8, 123). On the other side of the coin, we hope to boost the activity of anti-cancer immune cells, mainly Th1/CTL lymphocytes and NK cells, by facilitating antigen-presentation and T cell co-activation [22,105,106]. It is important to note that cancer promoting and inhibiting immune responses do not function in isolation, but cross-regulate each other within tumor stroma, further demonstrating the need to couple anti-inflammatory and T cell-activating agents for the treatment of cancers in the skin and other organs [7,8,37].

While the research on cancer vaccine and more recently T cell modulatory/co-stimulatory pathways has led to significant progress in the treatment of melanoma and other solid tumors [105,106], clinical trial on anti-inflammatory agents against cancers is lacking at this point [8,123]. Agents that inhibit inflammatory cytokine production, receptor binding or receptor signaling may prove useful in the treatment or even prevention of skin malignancies. Several types of agents should be considered for clinical development. These include anti-TNFα monoclonal antibody that has been shown to be effective in the treatment for human rheumatoid arthritis, psoriatic arthritis and IBD [124,125], humanized anti-IL-6R antibody used against rheumatoid arthritis, systemic juvenile idiopathic arthritis and Castleman’s disease [126], and IL-23 and IL-17A antibodies already found to be effective and non-toxic in the treatment of various chronic inflammatory conditions such as rheumatoid arthritis, ankylosing spondylitis, IBD and psoriasis [127,128,129,130,131,132]. It is important to note that chronic inflammatory molecules drive skin cancer development by signaling to both tumor cells and immune cells. In the case of cancer immune surveillance and immunotherapy, tumor-promoting cytokines also function as inhibitors against effective anti-cancer immune response [7,105]. It remains to be tested if the inhibition of proinflammatory cytokines can further improve the efficacy and/or safety of cancer immune therapies, such as checkpoint blockade therapies that have achieved significant improvement in the survival of patients with advanced melanoma [105,133].

## Competing Interests

The authors declare that they have no competing interests.
